# A new formula to predict the size and insertion depth of cuffed nasotracheal tube in children receiving dental surgery: a retrospective study

**DOI:** 10.1038/s41598-023-39793-0

**Published:** 2023-08-03

**Authors:** Chen-Hung Chou, Chia-Ling Tsai, Kai-Lieh Lin, Shao-Chun Wu, Min-Hsien Chiang, Hui-Wen Huang, Kuo-Chuan Hung

**Affiliations:** 1 Department of Anesthesiology, Kaohsiung Chang Gung Memorial Hospital, Chang Gung University College of Medicine, Kaohsiung, Taiwan; 2 Department of Pediatric Dentistry, Kaohsiung Chang Gung Memorial Hospital, Chang Gung University College of Medicine, Kaohsiung, Taiwan; 3 Department of Anesthesiology, Shin Huey Shin Hospital, Kaohsiung, Taiwan; 4 School of Medicine, College of Medicine, National Sun Yat-sen University, Kaohsiung, 804 Taiwan; 5 Department of Anesthesiology, Chi Mei Medical Center, No. 901, ChungHwa Road, YungKung District, Tainan, 71004 Taiwan

**Keywords:** Health care, Medical research

## Abstract

This retrospective study aimed to develop a new formula for selecting the appropriate size and determining the depth of the cuffed nasotracheal intubation (NTI) for a cuffed endotracheal tube (cETT) in pediatric patients undergoing dental surgery. In addition, the clinical data on cETT (i.e., the size and depth of insertion) was compared with those calculated with age-based formulas to evaluate their correlation. A total number of 684 patients who received NTI were enrolled (healthy group, n = 607; special-need group, n = 77). The ETT size used in real-world scenarios was smaller (i.e., about 0.5 and 0.94 mm) than the age-based formula, while the ETT depth was greater (i.e., about 1.5 cm) than the age-based formula in both groups. In the healthy group, age, gender, and body weight were identified as predictors of ETT size and depth through multiple linear regression analysis, while only age and body weight were predictors in the special-needs group. New formulas were developed based on these findings, with ETT size = 3.98 + 0.052 × age + 0.048 × gender (male = 1, female = 0) + 0.023 × body weight (kg) and ETT depth = 15.1 + 0.43 × age + 0.300 × gender (male = 1, female = 0) + 0.007 × body weight (kg). The new formula could be useful for both healthy and special-need pediatric populations undergoing dental procedures.

## Introduction

For children seven years or younger including those with special needs (e.g., cerebral palsy), dental treatment is often conducted under general anesthesia to avoid fear and anxiety because of separation from their parents^1,2^. Compared with sedation^3^, airway management with nasotracheal intubation (NTI) has become a common procedure to provide a safe and optimal oral surgical field during dental surgery^4,5^. For a successful NTI, anesthesiologists must be familiar with the techniques of the procedure, choice of an optimal size of nasotracheal tube (NTT) that matched the tracheal diameter^6^, and determination of the insertion depth of NTT to avoid airway-related complications. Regarding NTT size, a smaller size is associated with the risks of air leak, inadequate mechanical ventilation, and pulmonary aspiration, while a larger one may predispose the tracheal mucosa to ischemic damage that may contribute to postintubation croup and chronic subglottic stenosis^7^. Choosing the NTT of correct size is essential for the prevention of repeated intubation attempts, which increases the risks of NTI-related morbidity and mortality^7^. The depth of NTT insertion is also of utmost importance as an incorrect insertion depth may predispose to endobronchial intubation or inadvertent extubation^8^, especially in the pediatric population during intraoperative head-neck position adjustment^9–11^.

Despite these well-recognized NTT-related issues, choosing the optimal tube size and depth of intubation can be clinically challenging because of the variations in trachea dimensions and length with age and gender^12^. In addition to the concern of an increased airflow resistance and air leak due to the need for choosing an NTT of size smaller than that of the orotracheal tube to prevent nasal trauma and bleeding^13^, the short trachea in the pediatric population also pose significant difficulties for maintaining an appropriate distance between the vocal cords and the carina^4^. Although previous studies have attempted to envisage formulas that allow the calculation of tube size and insertion depth based on age, body weight, or both in the pediatric population^14–18^, most formulas were developed based on a single variable (e.g., age or body weight) from a limited number of patients with a relatively large range of ages^6,12^.

At present, there are no specific guidelines in the literature for selecting the appropriate size and insertion depth of NTT for children undergoing dental surgery. To fill this gap in knowledge, this study aimed to (1) assess the effectiveness of a previously proposed age-based formula^16,18,19^ for determining tube size and insertion depth in clinical practice, and (2) develop a new formula based on multiple predictors to improve the precision of selecting tube size and depth.

## Methods

### Ethics and study population

The current retrospective single-institution study was officially approved by the Institutional Review Board of Kaohsiung Chang Gung Memorial Hospital (IRB number: 202002537B0) and waived the requirement for informed consent due to the anonymity and retrospective nature of the study without research-oriented interventions or interactions with the subjects. In addition, all methods were performed in accordance with the relevant guidelines and regulations. Pediatric patients (< 18 years) receiving elective dental procedures (e.g., extractions, odontectomy, pulp therapy, and restoration) under sevoflurane-based general anesthesia with NTI from January 2017 to December 2019 were retrospectively reviewed to collect information about the size and insertion depth of NTT. Pediatric patients with a difficult airway, those who concomitantly received both dental and non-dental surgeries, and those in whom sevoflurane was not used as the agent for anesthesia maintenance (e.g., desflurane or total intravenous anesthesia) were excluded. In addition, pediatric patients who received oral tracheal tube or supraglottic airway as airway rescue devices were also excluded from our chart review. In the current study, children were divided into those with special health care needs (defined as having at least one type of physical or mental disability) (i.e., special-need group) and those with neither physical nor mental disabilities (i.e., healthy group) to explore possible differences in NTT size and insertion depth between the two groups.

### Theoretical and real-world standards for NTT size selection and depth of insertion

Although the number of attempts of intubation was not documented in the anesthetic records, all procedures were performed by 10 experienced attending anesthesiologists who had 5- to 20-year experience of pediatric dental anesthesia adept at pediatric nasotracheal intubation. At our institute, there was a standard operating procedure for anesthesia in children receiving dental procedures. Before anesthetic induction, the size of cuffed tubes (Medtronic Shiley™ Cuffed Basic Endotracheal Tubes) was determined by an age-based formula "internal diameter [mm] = 4 + [age (years)/4]"^19^. Nevertheless, the final choice of the size of NTT was entirely at the discretion of the anesthesiologist. Based on clinical practice, the size of NTT was considered too large if excessive resistance was encountered during tube insertion into the nostril or glottis, while the size was deemed too small when air bubbles were observed in the oral cavity after successful tracheal intubation and cuff inflation. The occurrence of either of these conditions required a change in size of NTT for reintubation.

With respect to the depth of NTT placement, the initial insertion depth was calculated by using the formula "tube insertion depth [cm] = [15 + age (years)/2]"^16,18^. Because radiographic identification of tube position^20^ was not routinely performed, the final insertion depth of NTT was confirmed with the following procedures. After chest auscultation to exclude the possibility of endobronchial intubation (i.e., too deep), the cuff ballotability method was used to verify the position of the cuff at the level of sternal notch^21,22^ according to the finding of a previous study demonstrating a 95% accuracy when comparing between a suprasternal notch position of the endotracheal tube cuff and the depth of insertion on chest radiograph^23^. After the NTT was secured in position, the cuff pressure was routinely adjusted using a manometer to maintain a cuff pressure between 20 to 30 cmH_2_O.

### General anesthesia induction and management

The anesthesia of all patients was induced by intravenous anesthetic agents (e.g., propofol 2–3 mg/kg or thiamylal 4–5 mg/kg) or 6% inhalational sevoflurane for those with difficult intravenous access, followed by intravenous cisatracurium at a dose of 0.1–0.3 mg/kg. After anesthetic induction and muscle paralysis, NTI was attempted with video laryngoscopy and Magill forceps. Following successful airway establishment, general anesthesia was maintained with inhalation agents (e.g., sevoflurane) while mechanical ventilation was set using pressure-controlled ventilation mode or pressure control ventilation-volume guaranteed mode. After anesthesia induction and successful NTI, prophylactic intravenous dexamethasone with a maximum dose of 5 mg was given for the prevention of nausea/vomiting and airway swelling at the anesthesiologist’s discretion. Maintenance of general anesthesia was achieved through inhalational sevoflurane within 1–2 minimal alveolar concentration (MAC) depending on the patient's vital signs. Intravenous atropine bolus at a dose of 0.01 mg/kg was administered for bradycardia. Intravenous fluid supplementation with 5% dextrose saline was given at a rate of 2–6 mL/kg/hr. After switching off the sevoflurane vaporizer at the end of dental surgery, the ventilator was shifted to manual support until return of spontaneous breathing. Reverse agents including intravenous neostigmine 0.05 mg/kg and atropine 0.02 mg/kg were used when spontaneous breathing occurs. Awake-extubation was considered when all of the following criteria were fulfilled: (1) an end tidal sevoflurane concentration less than 0.2%, and (2) the appearance of facial grimace, purposeful movement, conjugate gaze, and eye opening, as well as (3) a tidal volume greater than 5 mL/kg. After extubation, the pediatric patient was sent to the post-anesthesia care unit (PACU) for further medical care.

In the PACU, the patient was accompanied by one family member and a nurse who was responsible for immediate detection and management of adverse events including oxygen desaturation as well as the occurrence of airway obstruction, croup, laryngospasm, temperature instability, atelectasis, nausea, vomiting, agitation, neurological disturbance, postoperative pain, and surgical bleeding. The patient was ready to be discharged home or sent to the ward for further care after being monitored in the PACU for two hours and achieved a Modified Aldrete Score of more than 9 based on individual clinical condition.

### Data collection

Data on demographic profile (i.e., age, body weight, and gender), American Society of Anesthesiologists (ASA) physical status, tube size, depth of the NTT insertion, induction technique, perioperative anesthetic agent (e.g., sevoflurane consumption), anesthesia time, intravenous fluid volume, postoperative admission, and postoperative complication (e., sore throat, hoarseness) were collected.

### Primary and secondary outcomes

As there was no gold standard governing the choice of the size of NTT and the depth of insertion in the pediatric population, the primary outcome of the current investigation was the effectiveness of our newly developed predictive formulas for providing guidance on NTT application in real-world scenarios, while the secondary outcomes were the clinical differences in NTT size and insertion depth between the real-world data based on established clinical criteria and those derived from the conventional age-based formula.

### Statistical analysis

The demographic and clinical characteristics of healthy children and those with special needs were compared using independent sample t-test for continuous variables (e.g., age) or chi-square test for categorical variables (e.g., gender). Data on tube size and insertion depth in the real-world scenario were compared to those predicted by age-based formulas^16,18^ using paired sample t-test, which then identified potentially significant predictors for multivariable linear regression analysis. Multivariable linear regression analyses were conducted on the whole cohort, the healthy cohort, and the special-need cohort. Consequently, new formulas were derived from our retrospective data. To examine the correlation between the real-world data and those derived from our novel formula on NTT insertion depth prediction, a Bland–Altman analysis was performed^24^ with the satisfactory range of agreement being defined as mean bias ± 1.96 SD^25^. A *p* value < 0.05 was considered statistically significant. All statistical analyses were conducted using MedCalc version 20.019 (MedCalc Software Ltd, Ostend, Belgium).

## Results

### Study participants

**Figure 1:**
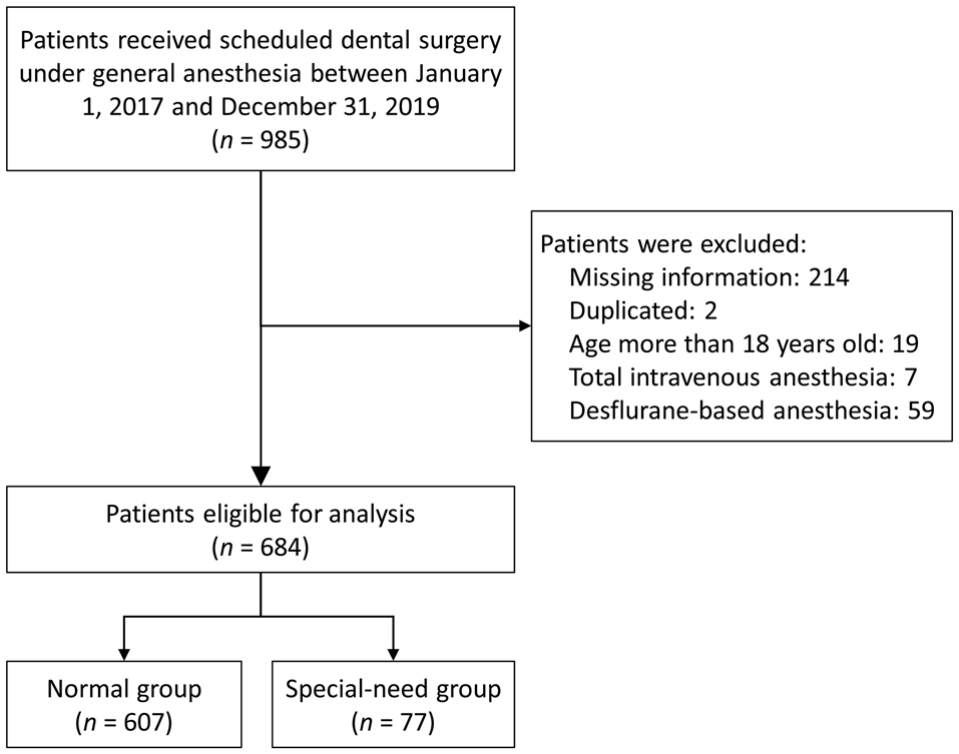
Flow diagram for study participants.

Chart review was performed on 985 children receiving elective dental surgeries under general anesthesia from January 2017 to December 2019. After the exclusion of 214 children with incomplete data, two children with duplicated records, 19 children with age over 18 years, seven children receiving TIVA for anesthesia maintenance, and 59 children with desflurane-based general anesthesia (Fig. 1), a total of 684 children were enrolled (healthy group, n = 607; special-need group, n = 77). No patients were excluded due to oral tracheal intubation or supraglottic airway insertion. No patients with ASA physical status IV or V were noted in our study.

### Demographics and clinical characteristics

Among the 684 enrolled patients, 77 were classified as the special-need group based on their medical diagnoses, while 607 patients were in the healthy group. The numbers of patients and their diagnoses in special-need group are summarized in Table 1. Compared with patients in the healthy group, those in the special-need group were older and heavier, requiring larger size of endotracheal tube and depth of intubation, having a higher ASA physical status and probability of postoperative admission, and more likely to receive intravenous induction (Table 2). No significant difference was observed in the remaining baseline characteristics between the two groups. The proportions of patients who received intravenous dexamethasone during anesthesia were 89.0% in the healthy group and 85.7% in the special-need group. Postoperative complications (e.g., sore throat, hoarseness, dyspnea) were not noted in both groups.

**Table 1:** Diagnosis of pediatric patients in the “special-need” group (n = 77).

Diagnosis	Case numbers (N)^a^
Epilepsy	17
Mental retardation/delayed development/learning deficit	26
Autism	16
Cerebral palsy	12
Congenital heart disease	7
Expressive language disorder	7
Rett’s syndrome	4
Alagille syndrome	1
Bilateral vesicoureteral reflux	1
Down’s syndrome	1
Adenylosuccinate lyase deficiency	1
Hemophilia A	1
Subclinical hyperthyroidism	1
Type I neurofibromatosis	1
Dysgenesis of corpus callosum with corpocephaly	1

^a^Listed by number with some patients having more than one diagnosis.

**Table 2:** Demographic and clinical characteristic of children.

	Total children (N = 684)	Healthy group (N = 607)	Special-need group (N = 77)	*P* value
Age (years)	5.1 ± 2.5 (range: 1–17)	4.7 ± 1.9 (range: 1–15)	7.6 ± 4.5 (range: 1–17)	< 0.001
Male gender	422 (61.7)	376 (61.9)	46 (59.7)	0.711
Body weight (kg)	19.1 ± 7.7	18.5 ± 6.5	23.8 ± 13.0	< 0.001
ASA				
ASA I	327 (47.8)	318 (52.4)	9 (11.7)	< 0.001
ASA II	338 (49.4)	285 (47.0)	53 (68.8)	
ASA III	19 (2.8)	4 (0.66)	15 (19.5)	
Mask induction	597 (87.3)	543 (89.5)	54 (70.1)	< 0.001
Opioid used	39 (5.7)	32 (5.3)	7 (9.1)	0.188
Dexamethasone (mg/kg)	0.11 ± 0.05	0.11 ± 0.05	0.10 ± 0.05	0.921
Anesthesia time (hours)	3.6 ± 1.2	3.6 ± 1.2	3.7 ± 1.2	0.258
Volume of fluid (ml/kg/h)	3.2 ± 1.1	3.2 ± 1.1	3.1 ± 1.2	0.435
Sevoflurane consumption (ml/kg/h)	0.97 ± 0.41	0.99 ± 0.40	0.78 ± 0.38	0.935
Postop admission	36 (5.3)	20 (3.3)	16 (20.8)	< 0.001
Postop complication	0 (0.0)	0 (0.0)	0 (0.0)	N/A
NTT size (mm)	4.7 ± 0.4	4.7 ± 0.3	5.0 ± 0.6	< 0.001
NTT insertion depth (cm)	18.8 ± 2.1	18.7 ± 2.0	20.2 ± 2.7	< 0.001

Data are given as number (%) or mean ± standard deviation.

*ASA* American Society of Anesthesiologists physical status, *NTT* nasotracheal tube, *N/A* not applicable, *Postop* postoperative.

### Theoretical and real-world data for NTT size selection and depth of insertion

Table 3 showed the comparison between the size and insertion depth of NTT in the real-world scenario and those calculated theoretically with the reported age-based formula^16,18,19^. For both groups, the size of real-world NTT was smaller than that suggested by the age-based formula (healthy group: 4.7 ± 0.3 mm vs. 5.2 ± 0.5 mm, respectively, difference −0.50 ± 0.35 mm, *p* < 0.001; special-need group: 5.0 ± 0.6 mm vs. 5.9 ± 1.1 mm, respectively, difference −0.94 ± 0.73 mm, *p* < 0.001). Therefore, the results showed that NTTs were actually half-size smaller than the size calculated from the formula for healthy children in the real-word scenario, while those used in the special-need group were close to one-size smaller compared to the size suggested according to the formula.

**Table 3:** Comparison with size and depth of nasotracheal intubation between clinical and recommendation data.

Outcome	Healthy group				Special-need group			
	Clinical	Recommendation^a,b^	Difference	*P* value	Clinical	Recommendation^a,b^	Difference	P value
ETT size (mm)	4.7 ± 0.3	5.2 ± 0.5	−0.50 ± 0.35	< 0.001	5.0 ± 0.6	5.9 ± 1.1	−0.94 ± 0.73	< 0.001
ETT depth (cm)	18.7 ± 2.0	17.4 ± 1.0	1.3 ± 1.6	< 0.001	20.2 ± 2.7	18.8 ± 2.2	1.4 ± 1.3	< 0.001

Data are given as frequency (%) or mean ± standard deviation.

*ETT* endotracheal tube.

^a^Recommendation of ETT size^19^.

^b^Recommendation of ETT depth^16,18^.

Regarding the insertion depth of NTT, the real-world insertion depth was about 1.5 cm deeper than that assessed with age-based formula in both groups (healthy group: 18.7 ± 2.0 cm vs. 17.4 ± 1.0 cm, respectively, difference 1.3 ± 1.6 cm, *p* < 0.001; special-need group: 20.2 ± 2.7 cm vs. 18.8 ± 2.2 cm, respectively, difference 1.4 ± 1.3 cm, *p* < 0.001).

### Development of a new formula guiding the choice of NTT size and intubation depth by multiple linear regression analysis

After conducting multiple linear regression analyses on data from both healthy and special-need groups, it was found that age, gender, and body weight explained 67.2% and 52% of the variance in NTT size and insertion depth, respectively (Table 4). In the healthy group, these demographic variables accounted for 57.5% and 42.1% of the variance in NTT size and insertion depth, respectively. However, in the special-need group, the percentages were notably higher at 84.3% and 83.7%, respectively. Using these results, two new formulas were created to calculate the size and insertion depth of NTTs, which are as follows: NTT size (mm) = 3.98 + 0.052 × age (years) + 0.048 × gender (male = 1, female = 0) + 0.023 × body weight (kg); NTT insertion depth = 15.1 + 0.43 × age (years) + 0.3 × gender (male = 1, female = 0) + 0.007 × body weight (kg) (Table 4).

**Table 4:** Multivariable linear regression analysis for size and depth of nasotracheal intubation.

Outcome/predictor	All			Healthy group			Special-need group		
	β (95% CI)	*P* value	R^2^	β (95% CI)	*P* value	R^2^	β (95% CI)	*P* value	R^2^
ETT size (mm)			0.672			0.575			0.843
Constant	3.98 (3.94–4.03)	< 0.001		3.99 (3.93–4.04)	< 0.001		3.98 (3.85–4.11)	< 0.001	
Age (year/old)	0.052 (0.043–0.062)	< 0.001		0.052 (0.040–0.065)	< 0.001		0.050 (0.032–0.069)	< 0.001	
Sex									
Female	0			0			0		
Male	0.048 (0.017–0.080)	0.010		0.051 (0.018–0.084)	0.024		0.027 (-0.082–0.137)	0.233	
Body weight (kg)	0.023 (0.020–0.026)	< 0.001		0.023 (0.019–0.026)	< 0.001		0.024 (0.018–0.030)	< 0.001	
ETT depth (cm)			0.520			0.421			0.837
Constant	15.1 (14.8–15.5)	< 0.001		15.0 (14.6–15.4)	< 0.001		15.3 (14.6–15.9)	< 0.001	
Age (year/old)	0.43 (0.36–0.50)	< 0.001		0.50 (0.40–0.59)	< 0.001		0.36 (0.27–0.46)	< 0.001	
Sex									
Female	0			0			0		
Male	0.30 (0.07–0.53)	0.010		0.29 (0.04–0.53)	0.024		0.33 (-0.22–0.89)	0.233	
Body weight (kg)	0.07 (0.05–0.09)	< 0.001		0.06 (0.03–0.09)	< 0.001		0.08 (0.05–0.11)	< 0.001	

Illustration: the new formula of pediatric nasotracheal intubation for all pediatrics.

NTT size = 3.98 + 0.052 × age + 0.048 × gender (male = 1, female = 0) + 0.023 × body weight (kg).

NTT depth = 15.1 + 0.43 × age + 0.300 × gender (male = 1, female = 0) + 0.007 × body weight (kg).

*ETT* endotracheal tube, *β* regression coefficient, *CI* confidence interval.

### Agreement between the new formulas and real-world data

**Figure 2:**
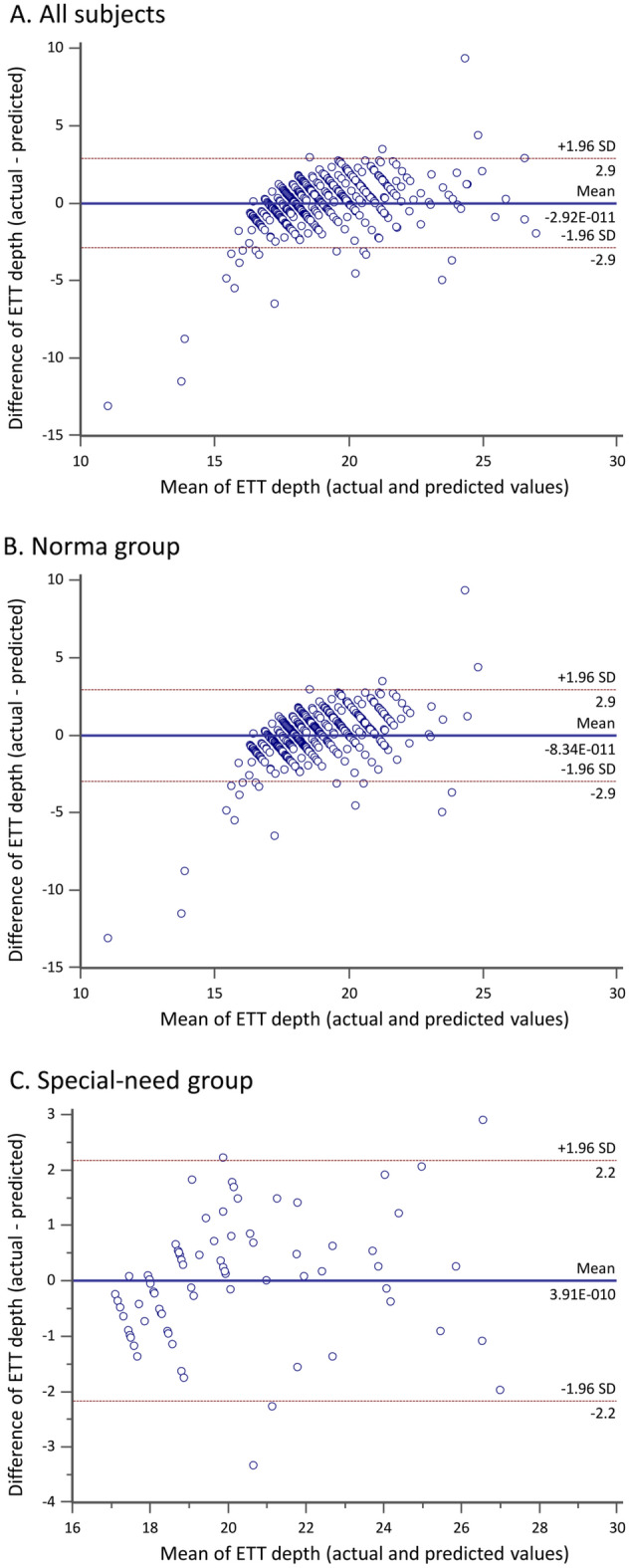
Bland–Altman plot showing the agreement between nasotracheal tube (NTT) depth predicted by the new formula and NTT depth recorded in real-world scenarios in (**A**) all subjects; (**B**) healthy group, and (**C**) special-need group. The solid line shows the mean value and the dotted line shows the range of 1.96 standard deviation (SD) of the values of bias.

Bland–Altman plots assessing the degree of agreement in insertion depth between the new formulas and real-world data showed that the mean bias (i.e., difference between the actual and predicted values) was close to 0 with the 95% limits of agreement lying within 2.9 cm in either all subjects (Fig. 2A) or in the healthy group (Fig. 2B). On the other hand, the mean bias was also close to 0 with a narrower 95% limits of agreement by 2.2 cm in the special-need group (Fig. 2C). A comparison of nasotracheal tube size and insertion depth between real-world data and data acquired from computation based on the newly developed formula in selected patients is shown in Table 5.

**Table 5:** Comparison of nasotracheal tube size and insertion depth between real-world data and data acquired from calculation based on a newly developed formula in selected patients.

	Case 1	Case 2	Case 3	Case 4	Case 5	Case 6
Predictor						
Age (year/old)	3	3	7	7	14	14
Sex	Female	Female	Male	Male	Female	Male
Body weight (kg)	12.5	12.5	20.0	20.0	34.0	33.3
Special need	No	Yes	No	Yes	Yes	Yes
NTT size (mm)						
Predicted value	4.42	4.44	4.85	4.84	5.51	5.52
Clinical value	4.5	4.5	5.0	5.0	5.5	5.0
NTT depth (cm)						
Predicted value^a^	17.3	17.4	20.0	19.8	23.1	23.4
Clinical value	14.0	17.0	18.0	20.0	25.0	22

*NTT* nasotracheal tube.

^a^Calculated with the new formula.

### Postoperative complications

Review of the medical records showed no postoperative complications such as nausea and vomiting, sore throat, hoarseness, dyspnea, choking, notable nasal bleeding/ epistaxis, wheezing, and intensive care unit admission in any patients. There were 20 patients (3.3%) in the healthy group and 16 patients (20.8%) in the special-need group who required postoperative admission. The reasons for postoperative admission were poor family support for postoperative dental care and a long commute time between home and hospital in the healthy group, while admission for observation or treatment was indicated for patients with a history of seizure, congenital heart disease, severe cerebral palsy with poor expectoration function, or necessity of further chest care in the special-need group.

## Discussion

In this large-scale study focusing on children subjected to dental procedures who received NTI for general anesthesia, a novel formula based on age, weight, as well as gender was developed and validated to predict the size and insertion depth of NTTs. To the best of our knowledge, this study is the first to investigate the tube size and insertion depth of NTT in children receiving dental procedures. Our results demonstrated that the insertion depth of NTTs was about 1.5 cm deeper in the real-world scenario compared to that calculated from the age-based formula [i.e., 15 + (age/2) cm] in children with or without special needs. For tube size selection, an NTT tube size (i.e., internal diameter) 0.5 mm smaller than that acquired with the age-based formula [i.e., 4 + (age/4)] mm was found to better fit the real-world situation in children without special needs. In contrast, NTTs with a 1 mm smaller size than that derived from the age-based formula may be required for children with special needs. Two novel formulas have been developed to compute the diameter and depth of insertion for NTTs., which are as follows: NTT size (mm) = 3.98 + 0.052 × age (years) + 0.048 × gender (male = 1, female = 0) + 0.023 × body weight (kg); NTT insertion depth = 15.1 + 0.43 × age (years) + 0.3 × gender (male = 1, female = 0) + 0.007 × body weight (kg).

The anatomical structures of the airway are age-dependent with changes in size, shape, and relative positions from the neonatal period to adulthood^26,27^. Furthermore, the development of children with special needs (e.g., Down syndrome) may be different from their healthy counterparts because of the existence of underlying diseases and altered nutrition status of the former^28^. Accordingly, the prediction of appropriate NTT size and insertion depth in children receiving dental procedures can be challenging regardless of their underlying conditions. Regarding the insertion depth of NTT, although chest radiography is considered the gold standard for determining the position of the tracheal tube relative to that of the carina^29^, the time-consuming nature and the need for radiation exposure especially in children are some of its downsides. Although several computational approaches based on age, body weight, or both are available to guide the choice of appropriate size and insertion depth of NTT [^14–18^, their accuracy remains controversial^30^. In fact, a previous study has reported disappointing rates of accurate insertion depth prediction as low as 66.9% and 44.4% for the age-based and height-based formulas, respectively^30^. One of the possible explanations for the low accuracy may be their dependence on a single variable for calculation.

One of the strengths of the current study was our focusing on children receiving dental procedures who invariably require general anesthesia for successful conduction of the operations. In addition, to the best of our knowledge, this study is the first to apply gender as a predictor to estimate the optimal tube size and insertion depth. Consistently, a previous study has underscored a gender influence on nasopharyngeal structure by showing larger anterior/posterior nasopharyngeal dimensions as well as a deeper nasopharynx airway in boys compared with girls aged between 3 and 16 years^31^. In addition, our finding of an actual insertion depth that was about 1.5 cm deeper than that assessed with the age-based formula in both groups was essential for general anesthesia in children whose head–neck movement during dental procedures may increase the risk of unexpected tube extubation or endobronchial intubation^9–11^. Our proposed formula may allow a quick assessment of the appropriate size and insertion depth of NTT, thereby minimizing the risk of accidental tube dislodgement or displacement.

Besides radiography and formula-based guidance, there are different approaches towards the determination of correct NTT position. Ultrasound, which is a non-radiographic method to verify the exact location of the endotracheal tube cuff after endotracheal intubation^32^, is a common clinical tool for evaluating tube position. A previous study reported a strong correlation between the ultrasound technique and fluoroscopy in locating the trachea tube^33^, suggesting reliability of ultrasound in this clinical setting. Another technique used in guiding ETT position is through palpation as in the cuff ballotability method^21,22^, in which the correct position of the endotracheal tube is considered when a distension of the ETT cuff can be felt over the suprasternal notch with one hand when pressure is applied to the pilot balloon with the other, and vice versa^21^. Because the identification of ETT cuff at the suprasternal notch is known to correlate with correct insertion depth on chest radiograph with an accuracy as high as 95% in the pediatric population^23^, the cuff ballotability method is considered the gold standard in determining ETT position in the real-world clinical setting. The current study of over six hundred pediatric patients showed the clinical applicability of the cuff ballotability method in terms of the ease of performance and the lack of associated complications (i.e., tube dislodgement).

Regarding tube size, the present study showed that the sizes of NTT in actual clinical practice were 0.5 mm and 1.0 mm smaller than those acquired based on the age-based formula^19^ for healthy children and those with special needs, respectively. Therefore, our finding, which would allow smoother tube passage through the nostril to avoid unnecessary soft tissue injury and bleeding that may contribute to airway complications, may be of particular importance for children with special needs who are more injury-prone because of their frequent association with mental and physical developmental disabilities^5^. Our finding was consistent with that of a previous study that proposed initial oral endotracheal intubation with an endotracheal tube at least two sizes smaller in children diagnosed with Down syndrome than that in their age-matched healthy counterparts^28^. Such knowledge is critical for the pediatric population taking into consideration that endotracheal intubation for two attempts was reported to correlate with a more than three-fold increased odds of adverse effects and over 4.5-fold for three or more attempts in a pediatric emergency care setting^34^. Therefore, our finding may provide guidance on the quick selection of an NTT tube of appropriate size to avoid repeated tracheal intubation in the pediatric population.

There were some limitations in our study. First, our prediction formula was based on children of the same ethnic group in a single-center setting; therefore, its usefulness in populations of different ethnicities may be limited. Second, because our proposed formula was derived from cuffed ETTs, it may not be applicable to uncuffed ETTs that may be associated with the concern of air leak. Third, although previous studies have highlighted the association between body height and tracheal dimension^35,36^, the lack of information about body height in most of the medical records precluded our incorporation of this parameter into our formula. Fourth, our results that were derived mostly from children less than ten years of age may not be extrapolated to those close to their adolescence and neonates. Fifth, our restriction of patients who only received sevoflurane-based general anesthesia to avoid the confounding effects of anesthetic-induced airway events (e.g., sore throat, nausea/vomiting)^37^ may reduce the number of eligible individuals for the current study. Finally, despite the precise prediction of ETT size with our proposed formula, the error associated with the predicted depth of intubation could be up to 2 cm (Table 5) that may be crucial for pediatric anesthesia practice. Therefore, although the proposed formula may guide the depth of intubation, confirmation of tube position with clinically validated approaches (e.g., cuff ballotability method or ultrasound) remains vital.

In conclusion, based on age, gender, and body weight, the current study proposed a new formula for guiding size selection of cuffed nasal endotracheal tubes and insertion depth in healthy children and those with special needs. Besides the novel identification of gender as a significant parameter in our formula, the derived results correlated more accurately with the real-world data than that acquired from previous age-based formula, thereby providing practical guidance to avoid potential airway complications and enhance anesthesia safety in the pediatric population.

## Data Availability

The datasets used and/or analyzed during the current study are available from the corresponding author upon reasonable request.
